# Use of an Autologous Platelet-Rich Concentrate in Hypospadias Repair: A Systematic Review and Meta analysis

**DOI:** 10.1080/2090598X.2022.2149129

**Published:** 2022-11-22

**Authors:** Nitinkumar Borkar, Charu Tiwari, Debajyoti Mohanty, Arvind Sinha, Vijai Datta Upadhyaya

**Affiliations:** a Department of Paediatric Surgery, AIIMS, Raipur, India; b Department of General Surgery, AIIMS, Raipur, India; c Department of Paediatric Surgery, AIIMS, Jodhpur, India; d Department of Paediatric Surgery, SGPGIMS, Lucknow, India

**Keywords:** Autologous platelet-rich concentrate, intermediate layer, hypospadias, surgery, outcome

## Abstract

**Background:**

There is unanimous agreement amongst hypospadias surgeons to use an intermediate layer to cover the neourethra. Dartos fascia and tunica vaginalis (TV) flaps are the most preferred tissues to be used. Tissue glue, sealants and biomaterials are also useful where there is a paucity of local tissue to cover the neourethra. But these blood-derived products have associated infectious and allergic risks. The autologous human platelet concentrate (APC) contains biologically active factors and is safe for wound healing and soft tissue reconstruction. It has been used by few surgeons as an intermediate layer in hypospadias repair. This systematic review and meta-analysis aim to systematically compare the outcomes of hypospadias surgery in children with or without using APCs.

**Methods:**

This systematic review and meta-analysis was conducted as per the Preferred Reporting Items for Systematic Review and Meta-Analysis (PRISMA) guidelines. Meta-analysis protocol was registered with INPLASY. A systematic, detailed search was carried out by the authors in the electronic databases, including Medline, Embase, CENTRAL, Scopus, Google Scholar and clinical trial registry. Studies were selected and compared based on primary outcome measures like urethra-cutaneous fistula, meatal stenosis, wound infection and operative time. Statistical analysis was performed using a fixed-effect model, pooled risk ratio and I^2^ heterogeneity.

**Results:**

Four randomized studies with a total of 355 patients were included. Pooled analysis for outcome of urethra-cutaneous fistula (UCF) showed no significant difference between the groups with APC and without APC. Pooled analysis for the other outcome like meatal stenosis, wound infection and total complications showed a decrease in incidence of these complications in groups with APC.

**Conclusion:**

This meta-analysis shows that there is a reduction in the incidence of wound infection, meatal stenosis and total complications in patients where APC was used to cover the neourethra, although no such difference was observed in UCF rates.

## Background

Hypospadias is the most common congenital anomaly of penis. It is the second most common genital birth defect in boys after cryptorchidism [1,2]. The incidence of hypospadias is 1 in 200 to 250 live male births [3–5]. The clinical spectrum of hypospadias is based on the location of the urethral meatus and associated chordee. More than 300 surgical techniques for hypospadias repair can be found in the literature [6].

Various techniques have been proposed for hypospadias repair involving different surgical disciplines; however, the unanimous agreement among them is to use an intermediate layer to cover the neourethra. The reinforcement of hypospadias repair with an intermediate layer is believed to reduce the incidence of postoperative complications. Medical Literature describes the use of various tissues such as de‐epithelised overlap skin flap, dartos fascia, corpus spongiosum, tunica vaginalis (TV) flap and tunica vaginalis graft to provide cover to neourethra [7–11]. Dartos fascia and tunica vaginalis flaps are the most preferred tissue to be used as an intermediate layer. It has been proved that the use of intermediate layer in hypospadias repair reduces the incidence of postoperative complications. Snow BW [10] was the first to introduce the use of TV as a blanket wrap to cover reconstructed neourethra. Tiwari et al. [11] reported that tunica vaginalis can also be used as a graft to cover neourethra. Though dissection of TV flap is easy and well vascularised, TV flap may sometimes be limited by its length to reach up to the apex of urethroplasty suture-line and will also not be available in re-do surgeries once used. Local dartos is considered to be more physiological, but it is limited to be used in redo surgeries or in staged surgeries. Tissue glue, sealants and biomaterials are useful in such conditions where there is a paucity of local tissue to cover the neourethra [12].

Tissue sealants have gained significant attention in the recent past. Covering the neourethra with tissue sealant during hypospadias repair provides additional coverage to the neourethra and reduces complications like urethra-cutaneous fistula (UCF) [12]. The major limitations of these blood-derived products are the associated infectious and allergic risks and the increased cost. The autologous human platelet concentrate is derived from a small volume of plasma containing biologically active factors. This product facilitates hemostasis, synthesis of new connective tissue and revascularization. Autologous platelet concentrate (APC) is a safe product for wound healing, soft tissue reconstruction and bone healing [13,14]. It was first used in hypospadias surgery for the closure of UCF by Soyer et al. [15]. Guinot A et al. [16] was the first to use an autologous platelet-rich fibrin membrane for urethroplasty coverage with encouraging results. El-Galley et al. [17] showed that the concentration of the epidermal growth factor is significantly lower in the foreskin of boys with hypospadias than in normal boys. Autologous platelet concentrate increases the local concentration of g^r^owth factors and cytokines, enhancing the healing process and reducing the incidence of postoperative complications [14]. As this product is derived from the patient, it has a low cost and reduced risk of infection and allergic reaction. Autologous platelet concentrates (APC) include materials such as platelet-rich fibrin (PRF), platelet-rich plasma gel (PRPG) and platelet-rich plasma (PRP) [18].

Over the last 10 years, many case series [19,20] and comparative studies [21–23] were published using autologous platelet-rich concentrate as a barrier layer or adjuvant to the barrier layer during urethroplasty. This systematic review and meta-analysis aim to systematically compare outcomes of hypospadias surgery in children with or without autologous platelet concentrates.

## Materials and Methods

This systematic review and meta-analysis was conducted as per the Preferred Reporting Items for Systematic Review/and Meta-analysis (PRISMA) guidelines. Protocol for this meta-ananlysis has been registered with INPLASY (Protocol-202290098).

Preliminary literature search was done in PubMed and Cochrane Central Register of Controlled Trials (CENTRAL) to confirm the absence of published meta-analysis on this topic. Detailed electronic searches were done in the electronic databases including Medline, CENTRAL, Embase, Scopus and Google Scholar by two authors (CT, NB) independently till 15 September 2022. Also, clinical trial registry (clinicaltrials.gov) and major conference proceedings were searched till 15 September 2022, with no language restrictions. Searches were rerun before the final analysis in case any further identified study could be included for analysis. The search terms used were (Autologous platelet rich plasma OR autologous platelet rich fibrin membrane OR Autologous platelet gel OR Platelet rich plasma OR PRP AND Hypospadias OR Hypospadias repair). Hand searches were also performed with related references’ lists in the identified studies.

## Eligibility Criteria

Participants/population – Operated hypospadias patients; Intervention – Autologous Platelet Concentrate to cover neourethra; Comparator – Tissue covers to neourethra. Inclusion criteria – Operated hypospadias patients in whom autologous platelet-rich concentrate is used to cover neourethra

Exclusion criteria were tissue glue, sealants, and acellular dermal matrix used to cover the neourethra.

Primary outcomes included were Urethro-cutaneous fistula, meatal stenosis, stricture, wound infection and mean operative time. We included all randomised studies or comparative studies for our meta-analysis. We included studies reported as full text or published as abstract only where sufficient data are available and unpublished data from completed studies, if available.

## Data Collection and Analysis

### Study selection

Two authors (VU and NB) independently reviewed the abstract and title of the identified articles. After removing any duplicates from the search results, full texts of the potentially eligible studies were retrieved. The authors independently assessed the full‐text articles to identify the eligible studies for inclusion. Any disagreement between the authors was resolved through discussion and a third author was consulted if consensus was not achieved. Excluded studies and the reasons for exclusion were documented. This selection process was presented in the PRISMA flow diagram. We measured the Cohen's Kappa coefficient for estimation of the interrater reliability for selecting potentially relevant studies.

### Data extraction

After selecting the relevant studies, two authors independently performed data extraction (CT, AS). Baseline information for each study (information of the author, year of publication), number of patients per study, number of patients in each group, mean/median age of the patients, age range, along with the outcome parameters mentioned above was extracted in a data extraction table using MS Excel (Version 16.16.27). Any discrepancies among the observers were resolved through consensus and in consultation with another author (NB).

### Methodological quality assessment

The methodological quality of the included studies was independently assessed by two authors (CT and DM) utilizing the modified Downs and Black scale [24]. This 27-item validated scale (score ranging from 0 to 28) was also adopted to include studies other than randomized controlled trials (RCTs) (Table 1). The measurement of the inter-rater reliability agreement was performed using kappa statistics. Based on the kappa values, the level of agreement was defined as almost perfect (0.81–1.00), substantial (0.6–0.80), moderate (0.41-.60), fair (0.21–0.40), slight (0.00–0.20) and poor (<0.00).

**Table 1.** Baseline characteristics of all included studies.

S/N	Studies	Setting	Study period	Design	Patients (N)	Mean age (months)	Type of hypospadias	Repair and intermediate layer	F/U (months)	Reported outcomes
1	Guinot et al. 2014	France	Prospective (PRP): June 2010 to September 2011 Retrospective (Control): 2008–2009	Bidirectional	Total 109; 33 in PRP group and 72 in Dartos group	8 months (range: 3.5–67 months)	Distal hypospadias	33 in PRP group and 72 in Dartos group Thiersch –Duplay with TIP	8 months (6–18 months)	Complication rate: UCF
2	Mahmoud et al. 2019	Egypt	October 2011 to December 2016	Prospective randomised	Total – 180 (90 in each group)	Range 12–65 months mean age 27.9 months	Subcoronal, distal and midpenile hypospadias	90 patient – TIP with PRP 90 patients – TIP with dartos flap	23.5 months (13–63 months)	UCF, partial glans dehiscence, meatal stenosis, urethral stricture, wound infection, operative time, cosmesis
3	Eryilmaz et al. 2020	Turkey	May 2019 to August 2019	Prospective randomised	Total – 40 (20 in each group)	6.85 ± 3.83 in PRP group 6.80 ± 3.89 in Dartos group	Mid penile hypospadias	20 patient – TIP with PRP and dartos flap 20 patients – TIP with dartos flap only	5 months	UCF, postoperative infection, urethral stenosis
4	Elsayem et al. 2021	Egypt	Jan 2018 to Jan 2020	Prospective randomised	Total – 30 (15 in each group)	5–24 months	All types of hypospadias including re-do who fitted for TIP repair	15 patient – TIP with PRP 15 patients – TIP with dartos flap	Not mentioned	UCF, meatal stenosis, infection, glans dehiscence, hematoma, bleeding, edema, skin necrosis

### Statistical analysis

Data were analyzed using Rev-Man 5.4. software. Continuous variables such as mean operative time were analyzed as mean differences with 95% confidence intervals (CIs). Dichotomous variables were analyzed as risk ratios (RRs) with 95% CIs.

The individual patient was the preferred unit of analysis in our study. Heterogeneity was identified by visual assessment of the studies’ confidence intervals in the forest plot (eyeball test). Heterogeneity was examined explicitly with I^2^ statistics. For quantifying the heterogeneity of the included studies, the following ranges of I^2^ statistics were used to guide the interpretation [25]:

0% to 40%: might not be important;

30% to 60%: may represent moderate heterogeneity;

50% to 90%: may represent substantial heterogeneity;

75% to 100%: considerable heterogeneity

## Results

### Study characteristics

**Figure 1.**
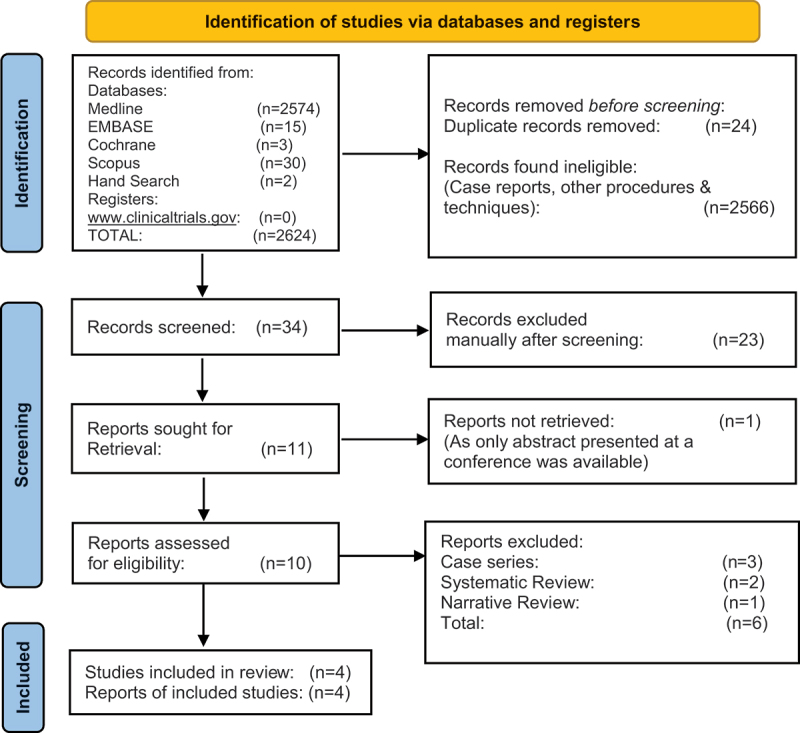
PRISMA flowchart depicting study identification and extraction.

The literature search yielded a total of 2624 studies. The Prisma flow diagram demonstrates our search and selection process (Figure 1). After removing the duplicate studies, 34 records were screened for title and abstract. A total of 11 articles were retrieved for full-text screening, following which only 4 studies [16,21–23] were included for the evaluation. The characteristics of the included studies are summarized in Table 1.

Four studies met our inclusion criteria, including 355 children (158 in the APC group and 197 in without APC group). Postoperative complications as reported in each of these four studies are summarized in Table 2.

**Table 2.** Summary Outcome Table.

SN	Study	Technique	Total (n)	UCF	MS	Stricture	PGD	PSWI/wound infection	Mean operative time (min)	Bleeding	Skin necrosis	Good cosmesis	Edema	Overall late complication rate
1	Guinot et al. [16]	With APC	33	2	-	-	-	-	Not mentioned	-	-	-	-	2
		Without APC	72	3	-	-	-	-	Not mentioned	-	-	-	-	3
2	Mahmoud et al. [22]	With APC	90	9	1	1	1	0	124.6 min	-	-	66	-	12
		Without PRP	90	12	1	1	4	6	136.4 min	-	-	62	-	24
3	Eryilmaz et al. [21]	With APC	20	2	1			1	Not mentioned	-	-	-	-	4
		Without APC	20	5	5			7	Not mentioned	-	-	-	-	7
4	Elsayem et al. [23]	With APC	15	0	4		1	4	Not provided	2	1	Not mentioned	2	-
		Without APC	15	3	8		3	5	But more in PRP gr	5	1	Not mentioned	4	-

UCF: urethrocutaneous fistula; MS: meatal stenosis; PGD: partial glans dehiscence; PSWI: partial superficial wound infection.

### Methodological Quality Assessment

Modified Down and Black scores were assigned to each study by two authors as depicted in Table 3. The score ranges from 17 to 27. The study by Elsayem K et al. [23] has the maximum score, and the study by Guinot A et al. [16] has the least score. There is a very high, positive correlation between the variables rater 1 and rater 2 with r = 0.97. Thus, there is a very high, positive association between rater 1 and rater 2 in this sample.

**Table 3.** Modified Down and Black scale scores for the included studies and inter-observer agreement (kappa statistics).

Study ID	Rater I	Rater 2	Kappa statistics
Guinot et al. [16]	17	16	0.97
Mahmoud et al. [22]	22	21	
Eryilmaz et al. [21]	19	17	
Elsayem et al. [23]	27	23	

## Meta-analysis of the outcome

### Urethro-cutaneous fistula (UCF)

**Figure 2.**
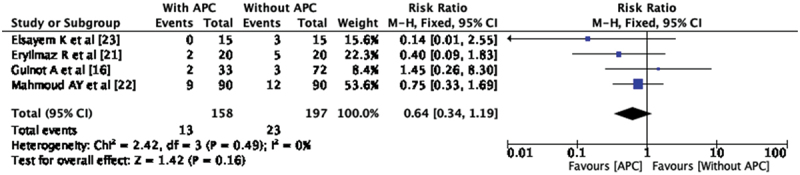
Forest plot – urethro-cutaneous Fistula.

**Figure 3.**
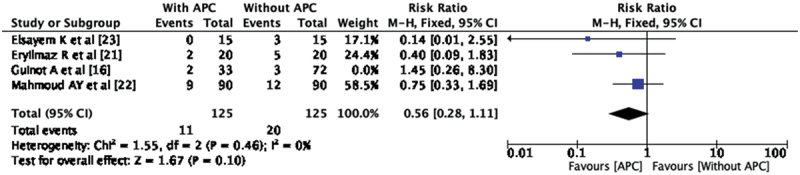
Forest plot – urethro-cutaneous fistula in TIP.

The postoperative complication, UCF has been reported in all the included studies. There are 13 UCF (8.38%) in APC group and 23 UCF (11.67%) in without APC group. Pooled analysis of the four studies showed no significant difference in the incidence of UCF among both groups (RR- 0.64, CI 0.34, 1.19). There was no statistical heterogeneity observed between the included studies (I^2^ = 0%) (Figure 2). Among all four studies included for the pooled analysis, Elsayem K et al. [23] used APC in re-do hypospadias repair also and other studies only in primary hypospadias repair. The subgroup analysis of these studies in which APC was used for the repair of primary hypospadias showed no statistically significant difference between two groups with RR-0.73 and CI 0.38, 1.40. Out of four included studies three studies [21–23] has used Snodgrass technique (TIP) for the repair of hypospadias. We performed subgroup analysis of three studies based on techniques of repair; it showed that there is no statistically significant difference between both groups in the incidence of urethra-cutaneous fistula (RR 0.56, CI 0.28, 1.11) (Figure 3). These are the three studies in which TIP was done; these are the similar studies which are RCTs included in meta-analysis.

### Meatal stenosis (MS)

**Figure 4.**
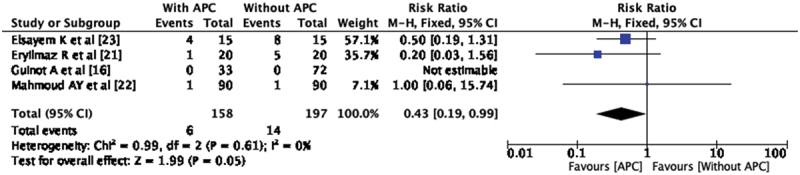
Forest plot – meatal stenosis.

Meatal stenosis has been reported in three out of four included studies. In the study by Guinot et al. [16], incidence of MS was not reported. There are six patients with meatal stenosis among 158 patients (3.79%) in APC group and 14 patients developed meatal stenosis among 197 patients (7.10%) in without APC group. Pooled analysis of all four included studies showed a statistically significant reduction in the incidence of MS in the group where APC was used during hypospadias repair (RR-0.43, CI 0.19, 0.99) (Figure 4). There was no heterogeneity observed between the studies (I^2^ = 0%). The pooled analysis for MS in all the patients repaired with the TIP technique in the three studies also yielded the same result, i.e. RR-0.43, CI 0.19, 0.99.

### Wound infection (WI)

**Figure 5.**
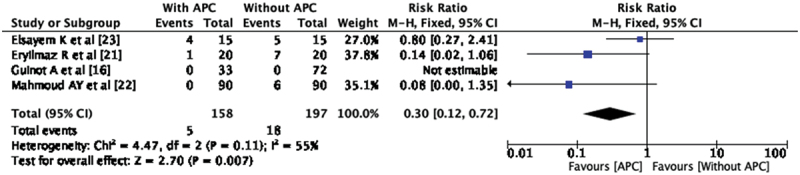
Forest plot – wound infection.

This complication was reported in three studies. In the study by Guinot et al. [16] there is no reported incidence of wound infection. There were five wound infections among APC group (3.16%) and 18 among without APC group (9.13%). Pooled analysis of all three studies shows a decrease in incidence of wound infection in APC group with statistical significance. (RR- 0.30, CI 0.12, 0.72) (Figure 5). There is moderate heterogeneity for this analysis I^2^ =55%.

### Glans dehiscence

Two of the included studies reported complication of glans dehiscence. In the study by Guinot et al. [16] and Eryilmaz R et al. [19], there is no reported incidence of glans dehiscence. Pooled analysis of the complication among the studies showed no significance between both groups (RR- 0.29, CI – 0.06, 1.32) without statistical heterogeneity I^2^ =0%.

### Total complications

The total complication rate was only reported by Mahmoud AY et al. [22]. In contrast,; the other studies did not report complications under the heading of total complication rate. When we added all the reported complications together to analyze the results, we observed that in the study by Elsayem K et al. [23], the total reported complications outnumbered the total number of included patients, possibly due to multiple complications arising in a single patient. We have excluded this study from the analysis. Pooled analysis of the other three studies showed a statistically significant reduction in the incidence of total complications among the patients where APC was used during hypospadias repair (RR-0.58, CI-0.39, 0.86).

Mean operative time was provided only by Mahmoud et al. [22], who reported a shorter duration of surgery in the PRP group; however, the difference was not statistically significant. Elsayem K et al. [23] have not specifically reported the mean operative time but mentioned that the mean operative time was more in the APC group. The rest two studies have not reported the mean operative time of the procedures.

## Discussion

This meta-analysis is the first comprehensive comparison of the incidence of postoperative complications in hypospadias repair with or without APC. Only four studies met the inclusion criteria set for our meta-analysis, involving a total of 355 children. The incidence of UCF in the APC group is 8.38%, while that in without APC group is 11.67%. Pooled analysis of the four studies showed no significant difference in the incidence of UCF among both groups without significant heterogeneity (RR- 0.64, CI 0.34, 1.19).

Pooled analysis of four studies for postoperative wound infection, meatal stenosis and total complication rates showed a decrease in the incidence of these outcomes in the APC group with statistical significance. There is no statistical difference in the outcome of glans dehiscence between the two groups.

More than 15% of children with hypospadias require more than two surgical procedures for a successful outcome [26]. Every hypospadiologist is well aware of the importance of the barrier (protective) layer, which can reduce the incidence of postoperative UCF. APC is well accepted as a barrier layer or an adjuvant to the barrier layer during urethroplasty. It has the advantage of not requiring healthy local tissue and also obviates the need for graft harvesting [27]. Platelet concentrates are a source of growth factors, including platelet-derived growth factor, vascular endothelial growth factor, fibroblast growth factor and cytokines. It can theoretically reduce complications like UCF, skin flap necrosis and wound infection by promoting wound healing. As a rich source of growth factors, APC is considered more advantageous over other adjuvant covering biomaterials in hypospadias repair.

The APC is obtained by centrifuging the patient’s blood and preserving the platelet-rich fraction [27]. APC can be obtained as a PRP membrane (sheet) with the addition of calcium chlorate to blood, as reported by Eryilmaz et al. [21] and Mahmoud et al. [22]. It can also be obtained as a platelet gel with the addition of calcium gluconate and thrombin to PRP as reported by Elsayem et al. [23] to cover the neourethra. Guinot et al. [16] obtained PRF, a second-generation platelet concentrate, by centrifuging blood at a lower speed. After centrifugation, a fibrin clot forms in the middle of the tube between the acellular plasma at the top and red blood cells at the bottom. The fibrin clot is first separated from the plasma and the red clot and then compressed between two surgical swabs to form a membrane. APC can be immediately prepared and utilized as the covering layer during the urethroplasty procedure. One of the significant advantages of these autologous products is the alleviation of allergic and infectious risks.

Apart from the comparative studies, many other studies have reported the use of APC in hypospadias repair. El-Sayed et al. [21] have reported the use of PRF in TIP repair in 20 patients. The incidence of UCF was 10% in their case series. Similarly, Al-Awadi et al. [20] have performed TIP repair using PRF in their case series of 30 patients with a 6.7% incidence of UCF. Both these studies have an incidence of UCF close to the pooled incidence of our meta-analysis, i.e. 8.38%. All our studies included patients with distal and mid-penile hypospadias, and APC was derived from the patient’s blood. A large volume of blood is required to derive APC to cover the neourethra in proximal hypospadias repair, which may not be possible with small circulating blood volume in young children.

This is the first systematic review and meta-analysis to compare the outcome of hypospadias repair using APC in terms of postoperative complications. The limitation of this meta-analysis is the inclusion of fewer RCTs and comparative studies for pooled analysis. Only four comparative studies were available for the pooled outcome analysis. Therefore, the results based on our pooled analysis should be used with caution due to the small number of available RCTs and comparative studies. The various outcomes reported by the studies were non-uniform; for example, cosmesis as an outcome measure was reported in only one study [22].

The patient population in our meta-analysis has only distal and mid-penile hypospadias. We could not find RCTs and comparative studies reporting APC use in proximal hypospadias repair. In one study, APC has been used as an additional layer overlying the dartos layer, which adds to the heterogeneity of the studies. We recommend well-designed RCTs with large sample sizes, including all types of hypospadias, to emphasize the benefit of the APC.

## Conclusion

This meta-analysis shows no significant difference in the UCF rates in patients undergoing hypospadias repair with and without APC. However, this meta-analysis shows a reduction in the incidence of wound infection, meatal stenosis and total complications in the patients where APC was used to cover the neourethra.
